# Predicting seasonal movements and distribution of the sperm whale using machine learning algorithms

**DOI:** 10.1002/ece3.7154

**Published:** 2021-01-12

**Authors:** Philippine Chambault, Sabrina Fossette, Mads Peter Heide‐Jørgensen, Daniel Jouannet, Michel Vély

**Affiliations:** ^1^ Greenland Institute of Natural Resources Copenhagen Denmark; ^2^ Megaptera Paris France; ^3^ Biodiversity and Conservation Science Department of Biodiversity, Conservation and Attractions Kensington WA Australia; ^4^ EXAGONE réseau TERIA Vitry‐sur‐Seine France

**Keywords:** cetacean, diving behavior, habitat modelling, *Physeter macrocephalus*, pseudo‐absences, SDM

## Abstract

Implementation of effective conservation planning relies on a robust understanding of the spatiotemporal distribution of the target species. In the marine realm, this is even more challenging for species rarely seen at the sea surface due to their extreme diving behavior like the sperm whales. Our study aims at (a) investigating the seasonal movements, (b) predicting the potential distribution, and (c) assessing the diel vertical behavior of this species in the Mascarene Archipelago in the south‐west Indian Ocean. Using 21 satellite tracks of sperm whales and eight environmental predictors, 14 supervised machine learning algorithms were tested and compared to predict the whales' potential distribution during the wet and dry season, separately. Fourteen of the whales remained in close proximity to Mauritius, while a migratory pattern was evidenced with a synchronized departure for eight females that headed towards Rodrigues Island. The best performing algorithm was the random forest, showing a strong affinity of the whales for sea surface height during the wet season and for bottom temperature during the dry season. A more dispersed distribution was predicted during the wet season, whereas a more restricted distribution to Mauritius and Reunion waters was found during the dry season, probably related to the breeding period. A diel pattern was observed in the diving behavior, likely following the vertical migration of squids. The results of our study fill a knowledge gap regarding seasonal movements and habitat affinities of this vulnerable species, for which a regional IUCN assessment is still missing in the Indian Ocean. Our findings also confirm the great potential of machine learning algorithms in conservation planning and provide highly reproductible tools to support dynamic ocean management.

## Graphical abstract

21 satellite tracked sperm whales in the south‐west Indian Ocean. The use of 14 machine learning algorithms predicted probabilities of the sperm whale's distribution during the wet and dry seasons.

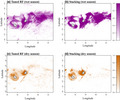

## 1 INTRODUCTION

Implementation of effective conservation planning relies on a robust understanding of the spatiotemporal distribution of the target species. In the marine realm, this is challenging for species that are rarely seen at the sea surface due to their extreme diving behavior such as beaked whales or sperm whales (Perrin et al., 2009). Among these deep diving predators, the sperm whale (*Physeter macrocephalus*) that can display long (~45 min) and deep dives (up to 1,860 m) with short surface intervals (~9 min) (Teloni et al., 2008; Watwood et al., 2006) is listed as vulnerable on the IUCN classification redlist. Depletion of this species’ global population was the result of excessive historic hunting and the current lack of complete recovery of the population worldwide (Whitehead, 2002). Although numerous studies have focused on sperm whales' spatial ecology and habitat selection (Gannier et al., 2002; Gannier & Praca, 2007; Jaquet, 1996; Pirotta et al., 2011, 2020; Watkins et al., 1999; Whitehead & Rendell, 2004), regional assessments are still limited to the north‐east coast of Europe and the Mediterranean Sea (Gannier et al., 2002; Laran & Drouot‐Dulau, 2007; Laran, Pettex, et al., 2017; Taylor et al., 2019; Virgili et al., 2019) despite the widespread occurrence of sperm whales in the Pacific (Davis et al., 2007; Whitehead et al., 2008) and Indian Oceans (Huijser et al., 2020; Laran, Authier, et al., 2017).

Since the establishment of the Indian Ocean Whale Sanctuary by the International Whaling Commission in 1979 (Holt, 1983), an increasing number of surveys focusing on the distribution of cetaceans (including sperm whales) in this region have been conducted (Laran, Authier, et al., 2017; Mannocci, Laran, et al., 2014). Recent aerial surveys conducted in the south‐west Indian Ocean confirmed the presence of sperm whales around Reunion and Mauritius Islands (Lambert et al., 2014; Laran, Authier, et al., 2017; Mannocci, Laran, et al., 2014), but in surprisingly low densities. Low densities may be the result of spatial aggregation of false absences (Virgili et al., 2017) due to deep divers like sperm whales spending a small amount of time at the sea surface, that is, 16%–21% (Hooker & Gerber, 2004; Jaquet et al., 2000). Although aerial surveys have significantly improved our understanding of the habitat use of marine megafauna in this region, this methodology can only provide a static picture of a species distribution unless surveys are regularly repeated throughout the year which is unlikely due to the cost of field campaigns and logistical difficulties (e.g., bad weather conditions). Satellite telemetry by tracking animals individually provides an alternative to assess deep divers’ movement patterns and fine‐scale habitat affinities through generating animal's trajectories in space and time. However, this approach only provides a small sample of the species distribution.

Distributed from polar regions to the equator, the sperm whale occupies a wide geographical range, but both sexes exhibit different distributions. While females inhabit mostly tropical and subtropical waters, adult males are mostly found at higher latitudes (except during the breeding season) in ice‐free deep waters or along the edges of continental shelves (Whitehead, 2018). After accompanying the females from 4 to 21 years, the young males can leave their female relatives to migrate towards higher latitudes (Christal et al., 2011). Although migrations of this species are not regular—accordingly poorly understood—north–south migrations have been evidenced in midlatitudes, but seasonal movements are less evident in tropical and subtropical regions (Whitehead, 2003). Both topographical (e.g., slope) and hydrological (e.g., eddies) factors have been shown to influence the distribution of sperm whales in the Mediterranean Sea (Cañadas et al., 2002; Gannier & Praca, 2007; Pirotta et al., 2011; Praca et al., 2009), the Atlantic (Biggs et al., 2000; Virgili et al., 2019; Waring et al., 2001), Pacific (Mannocci, Catalogna, et al., 2014), and Indian Oceans (Mannocci, Laran, et al., 2014).

Species distribution models (SDMs) have been largely used to predict potentially suitable habitats of marine species based on the relationships between the animal's occurrences and its environment (Austin, 2002; Elith & Leathwick, 2009). In conservation spatial planning, the potential distribution of a species is a powerful information tool to delineate protected areas in a more efficient way (Vierod et al., 2014). However, unlike the traditional regression methods commonly used in SDMs (e.g., GLM), machine learning‐based approaches have the ability to model complex polynomial relationships without relying on unrealistic assumptions (e.g., linearity) (Thessen, 2016). In contrast to classical methods, machine learning offers a wide range of algorithms to address ecological questions and to provide robust and accurate predictions. Machine learning is therefore a promising tool in species distribution modelling and conservation planning (Elith et al., 2006).

**FIGURE 1:**
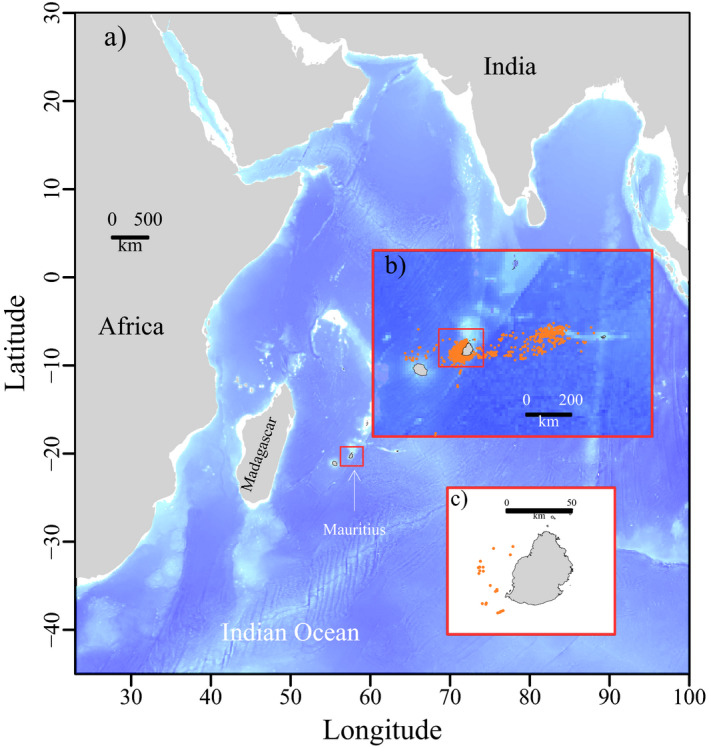
(a) Map of the study area located in the south‐west Indian Ocean. Panel (b) refers to Mauritius and Reunion Islands with the whales' locations in orange. Panel (c) to the tagging deployment locations along the west coast of Mauritius.

Using data from the first satellite tags (*n* = 21) deployed on sperm whales of both sexes inhabiting Mauritius waters (south‐west Indian Ocean, Figure 1), the predicted distribution of this deep diving species was modelled using a series of machine learning algorithms. By combining the individual satellite tracks with eight oceanographic variables (physical, surface, and subsurface predictors), our study aims at (a) investigating seasonal movements in the Indian Ocean, (b) predicting the potential distribution, and (c) assessing the diel pattern in sperm whale diving behavior. Since deep divers such as sperm and beaked whales might show a weak dependence on surface oceanographic characteristics (Mannocci, Laran, et al., 2014), we also included relevant subsurface covariates describing the vertical characteristics of the water column, that is, the mixed layer depth and the bottom temperature. These covariates are rarely taken into account in the habitat modelling of cetaceans. By combining machine learning, state‐of‐the‐art oceanographic variables, and the first tracking dataset around Mauritius, our results provide a first baseline needed to assess the spatiotemporal distribution of the vulnerable sperm whale in a poorly known region: the south‐west Indian Ocean (SWIO).

## 2 METHODS

### 2.1 Study area and tag deployment

Field work was conducted in the south‐western part of Mauritius Island in 2014, 2016, and 2018 (Figure 1). Sperm whales (*n* = 21) were instrumented with Wildlife Computers SPOT5, SPOT6, and SPLASH10 satellite transmitters (http://wildlifecomputers.com), modified for deployment and use on whales by Mikkel Villum Jensen (http://mikkelvillum.com). The tags were deployed using the ARTS, a modified pneumatic air gun, at about 8–10 m from the whale set at pressure of 11 bars (Heide‐Jørgensen et al., 2001). This is a standard procedure commonly used in tracking projects of large whales (Andrews et al., 2019). Both transmitters consisted of a stainless‐steel cylinder (SPOT5: 22 × 110 mm SPLASH10 24 × 155 mm) that contained the electronics and one lithium AA cell. A 38‐mm stop plate mounted 3 cm from the rear end of the tag stopped the tag at the surface of the skin and prevented the tag from penetrating deeper into the blubber/muscle layer. The rear end of the steel tube had an antenna (160 mm length) and a salt water switch that ensured that transmissions were only conducted when the rear part of the tag was out of the water. A pressure transducer was positioned just below the stop plate on SPLASH10 tags. In the front, the tags were equipped with a stainless‐steel anchor spear with a sharp pointed triangular tip and foldable barbs (40–50 mm) to impede expulsion from the blubber–muscle layer. The total length of the SPOT5 and SPOT6 from the stop plate to the tip of the anchor was 170 mm, and the mass of the instrument with attachment spear was 133 g. The total length of the SPLASH10 tag was 215 mm, and the mass of the instrument with attachment spear was 250 g.

The SPLASH10 tags collected summarized dive data in bins where dives to different depths and time spent at the same depths were binned into 100, 200, 300, 400, 500, 600, 700, 800, 900, 1,000, 1,100, 1,200, 1,300, >1,300 m bins. The duration of dives was summarized in these bins: 5, 10, 15, 20, 25, 30, 35, 40, 45, 50, 55, 60, 65, and >65 min. In addition to that, the maximum depth of dives was recorded for each 24 hr.

The tags were programmed to make a maximum of 250 transmissions per day between 04:00 and 16:00. The SPOT5 and SPOT6 tags were allowed to transmit every day in November through January and every other day the rest of the year. The SPLASH10 tags were allowed to collect dive data and transmit every day.

The tagging operation in Mauritius was conducted from a rigid hull inflatable boat (24 ft) with a 2 × 90 hp outboard motor, a steering panel, and at a maximum speed of 24 knots. The boat was equipped with a barrel to secure the tagger and provide a stable platform when approaching and tagging the whales. The satellite tags were deployed into the left or right flank of the whales about 1–2 m ahead of the dorsal fin and within 2 m from the midline of the whale's body. Approximate length of the tagged whales was estimated by comparing the size of the whale with the length of the boats involved with the tagging. Based on dimorphic morphology and on the Mauritius Marine Conservation Organization (M2CO) photo ID catalogue, mature males and mature females were also identified (Sarano & Sarano, 2017).

### 2.2 Location data processing

Location and dive data were obtained through the Argos Data Collection and Location System using the Kalman filter which greatly improves the location data (Lopez et al., 2014). Dive data were decoded in Wildlife Computers portal. All statistical analyses were performed using R software version 4.0.2 (R Core Team, 2020). We restricted our dataset to positions associated with a travel speed lower than 7 km/h (Wahlberg, 2002). Locations on land were also discarded. Any individuals containing less than 10 locations (for each season) were also discarded from the analysis. In order to assess seasonal patterns and monsoon periods of the Indian Ocean, seasons were classified as follows: dry season from April to November and wet season from December to March.

### 2.3 Kernel density estimation

To investigate the residency pattern of the sperm whales and locate their high‐use areas, a kernel utilization density approach was used for each season separately (Worton, 1989). To prevent over and under‐smoothing commonly found in kernel density estimations, we used a visual ad hoc procedure previously applied to terrestrial animals (Berger & Gese, 2007; Jacques et al., 2009) and recently tested in sea turtles (Chambault et al., 2020). The reference bandwidth parameter *h_ref_* was first calculated for each season. Then, *h_ref_* was sequentially reduced in 0.10 increment (0.9 *h_ref_*, 0.8 *h_ref_*, 0.7 *h_ref_*, etc.) until 0.1 *h_ref_*, and the most appropriate smoothing parameter was chosen visually by comparing the kernel density to the original location data (Kie, 2013). The core and global home ranges were calculated from the 50 and 90% kernel contours, respectively, for each season.

### 2.4 Environmental data

Strong relationships exist between cetaceans distribution and dynamic environmental variables (Mannocci, Catalogna, et al., 2014), such as *sea surface temperature* (SST), *sea surface height* (SSH), *ocean currents* (*U* and *V* components), and *ocean current velocity*. These variables were therefore tested as potential drivers of sperm whales' movements and to predict their potential distribution in the SWIO. In addition to surface variables, the *mixed layer depth* (MLD) was also considered as this variable is known to be closely related to primary productivity. The deep diving behavior of sperm whales might also be influenced by temperatures at the bottom of the water column where they mainly forage. Consequently, *bottom temperature* was also considered a likely driver of sperm whales' movements. Bathymetry was also extracted from GEBCO at a spatial resolution of 1 km, and the slope was subsequently derived from the bathymetry and expressed in degrees to get a proxy of the seafloor roughness. The dynamic variables were extracted monthly from the products *Global Ocean Physics Reanalysis Glorys S2V4* (PHYS 001‐024) and the *Global Ocean Physics Reanalysis Glorys12v1* (PHY‐001‐030) at a resolution of 0.08° (from E.U. Copernicus Marine Service Information). All variables were then set to the same spatial resolution of 0.08 decimal degree. Monthly grids of each predictor were then averaged for each season: between December and March for the wet season, and between April and November for the dry season.

### 2.5 Species distribution modelling

To identify the environmental drivers of sperm whale movement and predict their potential distribution, we built a series of species distribution models (SDMs) using multiple algorithms from the *caret* package in R. The aim was to relate the individual occurrences (observations provided by the tracking data), to the environmental predictors selected. We first used an environmental background‐based technique to generate pseudo‐absences (Hattab et al., 2017; Iturbide et al., 2015; Schickele et al., 2020; Senay et al., 2013), relying on the assumption that true absences are more likely located in areas that are environmentally dissimilar from presence locations. A principal component analysis (PCA) was used to generate a two‐dimensional environmental background representing the ordination results of the eight environmental variables available over the study area. One PCA was performed for each season separately. Pseudo‐absences were then randomly generated outside environmentally favorable areas for each season and in equal number to the filtered occurrences (e.g., tracking locations). To assess models’ sensitivity to the pseudo‐absences generation procedure, 10 different sets of pseudo‐absences were simulated (i.e., 10 runs for each season) for each algorithm. The eight environmental variables were then extracted at each occurrence and pseudo‐absence.

In order to find the most adequate model to predict the distribution of sperm whales with the highest accuracy, we tested 14 different algorithms belonging to the following categories:

1. Ensemble: random forest (RF) and stochastic gradient boosting (GBM);
2. Regression: generalized additive model (GAM) and multivariate adaptive regression splines (MARS);
3. Bayesian: Naïve‐Bayes (NB) and Bayesian additive regression trees (BayesGLM);
4. Decision tree: Logistic model trees (LMT) and C5.0;
5. Instance‐based: K‐nearest neighbors (KNN) and KKNN;
6. Dimensionality reduction: linear discriminate analysis (LDA) and quadratic discriminant analysis (QDA);
7. Support Vector Machine (SVM): SVM with radial kernel (SVMradial) and SVM with linear kernel (SVMlinear).

The 14 algorithms were ran for each simulation run using the presence of sperm whales (1: presence vs. 0: pseudo‐absence) as a response variable. The 14 models included the eight predictors mentioned above. All predictors were scaled between 0 and 1, and collinearity was checked using the variance inflation factor (below four). The dataset of each run was first randomly split between the training dataset (80% of the data) and the validation dataset (20% of the data). Each algorithm was run on the training dataset, while model evaluation was performed on the validation dataset. Model comparison was based on a tenfold cross‐validation with three repetitions using the following performance metrics calculated for each run on the 20% validation dataset: accuracy, kappa, sensitivity, specificity, true skill statistics (TSS), and F1 score. The best selected model was then tuned by testing several values of the *mtry* argument (the number randomly selected predictors). The “tuned model” was then used to generate ten prediction maps of the sperm whale's distribution (for each of the ten runs) and for each season separately. In parallel, the *caretEnsemble* package was used to generate ten predictions based on the combinations of the 14 algorithms previously tested, hereafter called the “stacking method.” The ten prediction maps of each approach were finally averaged to provide a final map of the potential distribution of sperm whales during the wet and dry season separately. The coefficients of variation were also calculated to provide a map of uncertainty (ratio of the standard deviation over the mean).

## 3 RESULTS

### 3.1 General tracking data

Across the 21 individuals equipped in Mauritius waters, three were males (#3963a, #20166b, and #50681b) and all remaining whales were females. The number of locations recorded per sperm whale ranged from 7 to 176 (#50678 vs. #93106, respectively)—Table 1. The tracking duration was on average 34 ± 20 days (range: 4–109 days). The total distance travelled varied between 154 (#6337a) and 3,112 km (#24642), and the average horizontal speed was 2.7 ± 0.3 km/h.

**TABLE 1:** Summary of the horizontal movements of the 21 sperm whales satellite tracked from Mauritius during the wet and dry seasons. Note that the whales with less than 10 locations per season were excluded from the habitat modelling analysis

PTT	Instrument	Sex	Start date	End date	Nloc	Duration (days)	Distance (km)	Speed(km/h)	Season
3963a	SPLASH10	Male	03/12/2014	19/01/2015	163	47	1,553.6	2.7	Wet
3965a	SPOT5	Female	02/12/2014	09/01/2015	110	38	1,397.7	2.9	Wet
20158a	SPOT5	Female	01/12/2014	05/01/2015	77	35	1,760.9	3.1	Wet
20160a	SPOT5	Female	01/12/2014	21/12/2014	50	20	825.03	3.0	Wet
20166b	SPOT5	Female	02/12/2014	24/12/2014	107	22	912.78	2.9	Wet
24642	SPOT6	Female	01/12/2018	18/03/2019	171	107	3,066.6	2.8	Wet
26712	SPOT6	Female	01/12/2018	30/12/2018	74	29	1,398.9	3.2	Wet
26715	SPOT6	Female	01/12/2018	17/12/2018	27	16	497.33	2.2	Wet
27261	SPOT6	Female	01/12/2018	23/01/2019	105	53	2,064.5	3.2	Wet
50678b	SPLASH10	Female	01/12/2018	27/12/2018	83	26	1,308.5	2.8	Wet
50681b	SPLASH10	Male	02/12/2018	02/12/2018	2	1	251.85	3.2	Wet
50682b	SPLASH10	Female	01/12/2018	28/12/2018	86	27	1,353.2	3.7	Wet
50683b	SPLASH10	Female	01/12/2018	03/12/2018	4	2	99.2	2.2	Wet
7618	SPOT6	Female	03/12/2018	20/01/2019	132	48	2,115.5	3.0	Wet
7926	SPOT6	Female	01/12/2018	21/01/2019	118	51	2,437	3.4	Wet
					*87 ± 40*	*35 ± 18*	*1,403 ± 611*	*2.9 ± 0.3*	
37282	SPLASH10	Female	04/05/2016	10/06/2016	98	37	1,275.3	2.6	Dry
50678	SPLASH10	Female	05/05/2016	09/05/2016	13	4	149.83	1.8	Dry
50681	SPLASH10	Female	03/05/2016	05/06/2016	106	33	1,403.9	2.9	Dry
50682	SPLASH10	Female	02/05/2016	23/05/2016	107	21	1,128	3.3	Dry
50683	SPLASH10	Female	03/05/2016	09/05/2016	26	6	222.16	2.7	Dry
93106	SPLASH10	Female	01/05/2016	08/08/2016	176	99	2,517.4	2.3	Dry
24642	SPOT6	Female	29/11/2018	30/11/2018	5	1	45.903	2.1	Dry
26712	SPOT6	Female	30/11/2018	30/11/2018	2	1	0.048959	0.0	Dry
27261	SPOT6	Female	30/11/2018	30/11/2018	5	1	52.516	2.8	Dry
50678b	SPLASH10	Female	24/11/2018	30/11/2018	33	6	229.93	2.9	Dry
50681b	SPLASH10	Male	24/11/2018	29/11/2018	19	5	212.85	2.6	Dry
50682b	SPLASH10	Female	29/11/2018	30/11/2018	13	1	60.563	3.1	Dry
50683b	SPLASH10	Female	24/11/2018	28/11/2018	9	4	183.44	1.7	Dry
7618	SPOT6	Female	25/11/2018	30/11/2018	26	5	149.53	1.8	Dry
7926	SPOT6	Female	29/11/2018	30/11/2018	8	1	52.53	2.4	Dry
					*43 ± 42*	*15 ± 17*	*512 ± 570*	*2.3 ± 0.6*	

Note

The numbers in italics are the means ± *SD* for each season.

### 3.2 Seasonal movements

**FIGURE 2:**
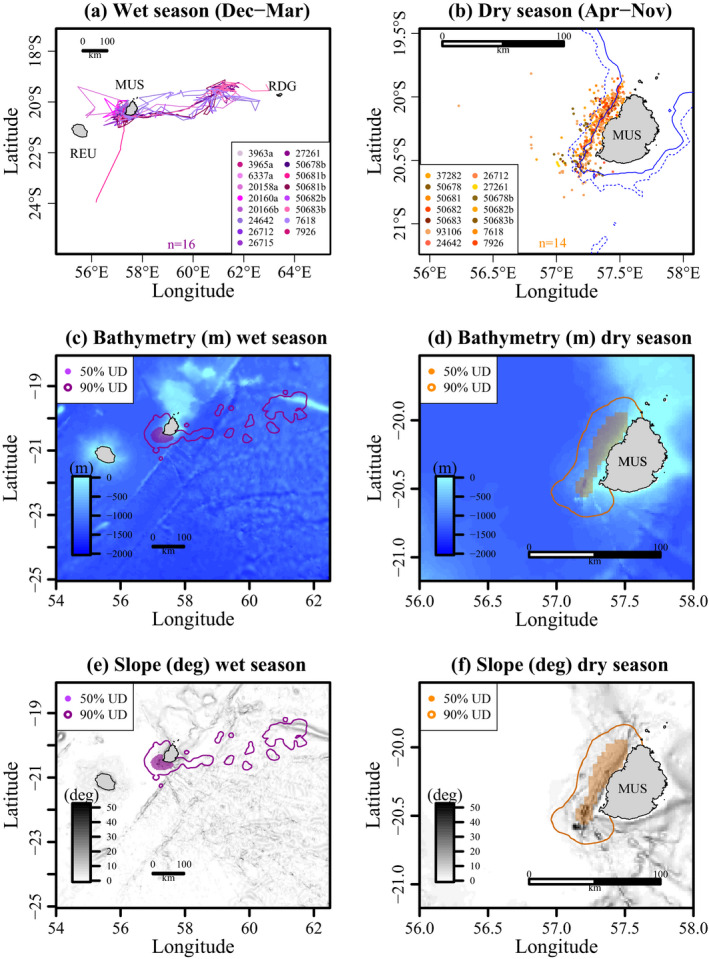
Locations of the sperm whales during the (a) wet and (b) dry seasons. (c,d) Maps of the bathymetry (expressed in m) and (e,f) the slope (expressed in degrees) over the study area. The utilization distributions (50% and 90% contours) were superimposed for the wet (left panel) and dry (right panel) seasons. MUS refers to Mauritius Island, REU to Reunion Island, and RDG to Rodrigues Island

A seasonal pattern in the movements of the whales was observed between the wet and dry seasons. Sixteen individuals were observed during the wet season from December to March (Figure 2a), and the tracks of 14 whales were available during the dry season from April to November (Figure 2b). The male tracked in 2018 (#50681b) was the only one migrating southward (Figure 2a). The kernel densities showed a core of activity in shallow waters near Mauritius for both seasons. During the wet season, the core home range (50% kernel contour) was located south‐west of Mauritius (Figure 2c). During the dry season, the resident whales exhibited strong site fidelity by inhabiting shallow waters along the west coast of the island (Figure 2d). The core home ranges were also located on variable topography for both seasons, for example, high slopes (Figure 2e,f).

### 3.3 Migratory patterns

**FIGURE 3:**
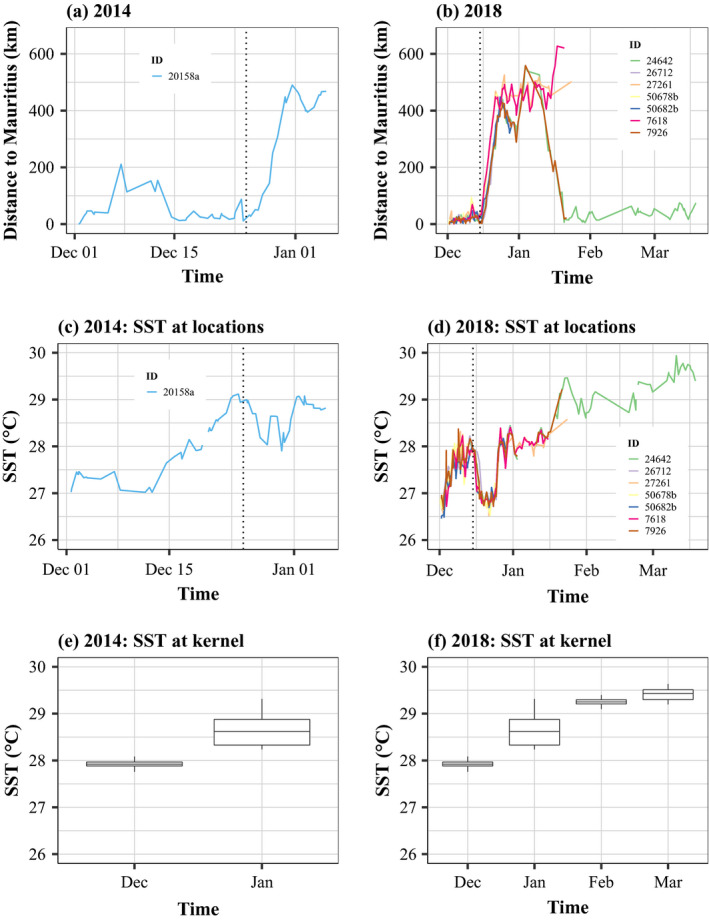
(a, b) Distance to the tagging site over time for the eight migrant whales tracked in 2014 and 2018. The vertical dotted lines refer to the departure date: 25 December 2014 in (a, c) and 15 December 2018 in (b, d). (c, d) SST extracted at the whale's locations in 2014 and 2018. (e, f) Box plots of the SST extracted inside the kernel 50% during the wet season in 2014 and 2018

Among the 16 individuals tracked during the wet season, eight whales (#20158a, #24642, #26712, #27261, #50678b, #50682b, #7618, and #7926) left Mauritius waters in December heading towards Rodrigues Island (Figures 2a and 3). Except one, these individuals were all mature females (based on Photo ID, morphology, size, and mother–calf association) and showed a synchronized departure from Mauritius. They made a loop eastward of Mauritius before returning at different times. In 2014, only one individual was considered migrant (departure on the 25 December 2014), while in 2018, seven whales initiated their short migration on the exact same date (15 December 2014). The increased distance from Mauritius matched the decrease in SST (extracted at the whales' positions) for all migrant whales (Figure 3c,d). Unlike the decrease in SST values at the whale's locations between December and January, the SST extracted inside the 50% kernel increased for both years from December to January (Figure 3e,f).

### 3.4 Algorithms comparison

**FIGURE 4:**
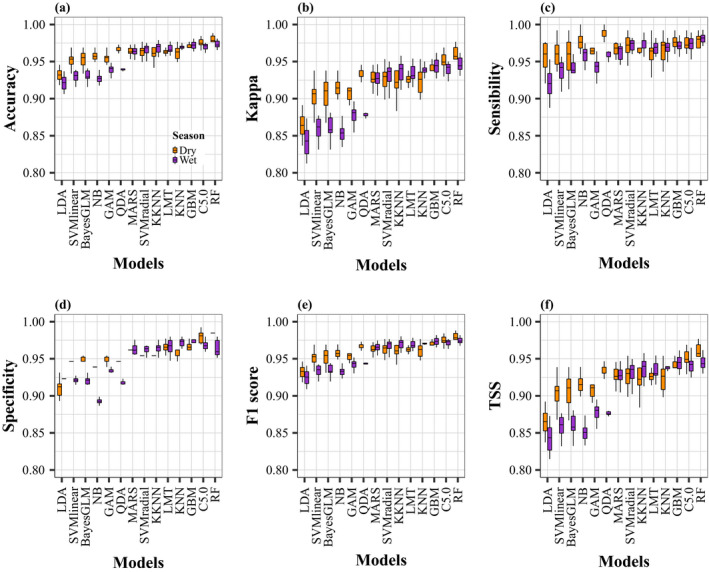
Box plots of the six performance metrics calculated for each of the 14 models and for each season. The values of each box plot include the performance metrics of each of the 10 simulation runs

The variation of the performance metrics across the ten simulation runs is illustrated by the box plots in Figure 4. Indeed, the small range of each box plot indicated little sensitivity to pseudo‐absence generation across the ten runs for both seasons. The mean values of the six performance metrics (accuracy, kappa, sensitivity, specificity, F1 score, and TSS) calculated from the tenfold cross‐validation were high (mean range: 0.81–0.99) for the 14 algorithms for both seasons (Figure 4), showing good predictive performance. Based on the six performance metrics, the best model was the random forest (RF) for both seasons, with values ranging from 0.93 to 0.99.

**FIGURE 5:**
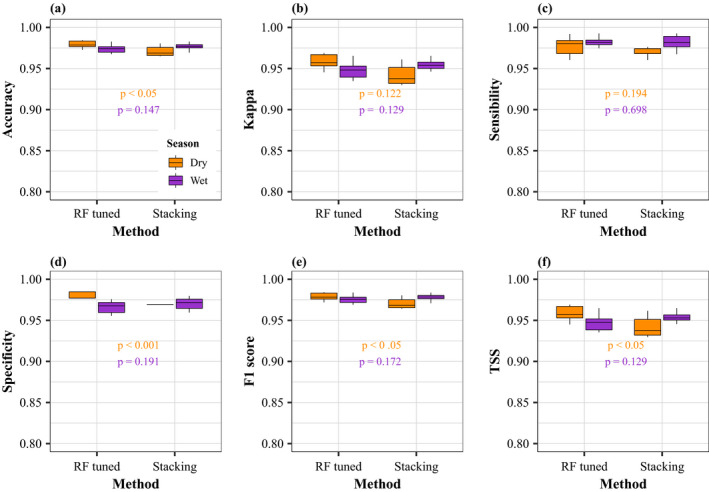
Box plots of the six performance metrics calculated for the tuned random forest model (RF tuned) and the stacking method for each season. The values of each box plot include the performance metrics of each of the 10 simulation runs

When comparing the tuned RF and the stacking method during the dry season, the tuned RF approach had slightly but significantly higher performance metrics compared to the stacking for all metrics: accuracy (mean: 0.978 vs. 0.970), specificity (mean: 0.980 vs. 0.970), F1 score (mean: 0.978 vs. 0.970), and TSS (mean: 0.957 vs. 0.941) (Kruskal–Wallis test, *p* < 0.05, Figure 5a,d,e,f). However, no significant difference was observed between both methods for the wet season (Kruskal–Wallis test, *p* > .05, Figure 5). Given such low differences, both methods were used to generate predictions of the whales' potential distribution for the wet and dry season separately.

### 3.5 Predicted distributions

**FIGURE 6:**
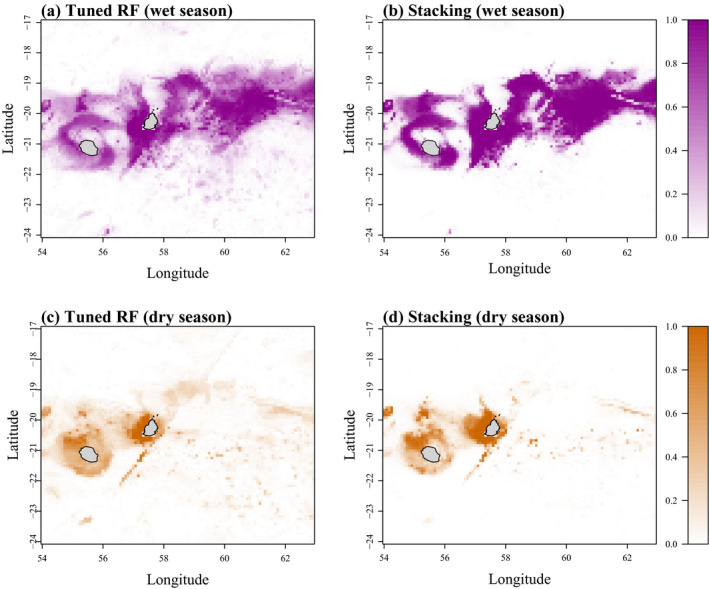
Averaged prediction maps of the sperm whales' probabilities during the (a, b) wet and (c, d) dry seasons calculated from the tuned random forest model (a, c) and the stacking method (b, d). 0 indicates a very low probability to see a whale and 1 a high probability to see a whale

The maps of the predicted distributions of the sperm whales reflected a pronounced seasonal pattern (Figure 6). Small differences were observed between both approaches, with similar spatial patterns and globally higher probabilities for the stacking method. During the wet season, the potential distribution was widely spread around Mauritius and between 59 and 62°E, which coincides with the migration of the eight individuals that left the coastal areas of the island (Figure 6a,b). In contrast, the favorable habitats during the dry season were mostly confined to areas close to Mauritius (mostly west and south‐west) and also north of Reunion Island. Some high probabilities of sperm whale presence were also identified on steep sloping habitats during both seasons, that is, south of Mauritius (Figure 6c,d). The most important covariates were the SSH and bottom temperature for the wet and dry season (for the tuned RF), respectively (Figure S1). The coefficients of variation were globally low (<3.2%), confirming the low variability between the ten simulations for both approaches (Figure S2).

### 3.6 Diving behavior

**FIGURE 7:**
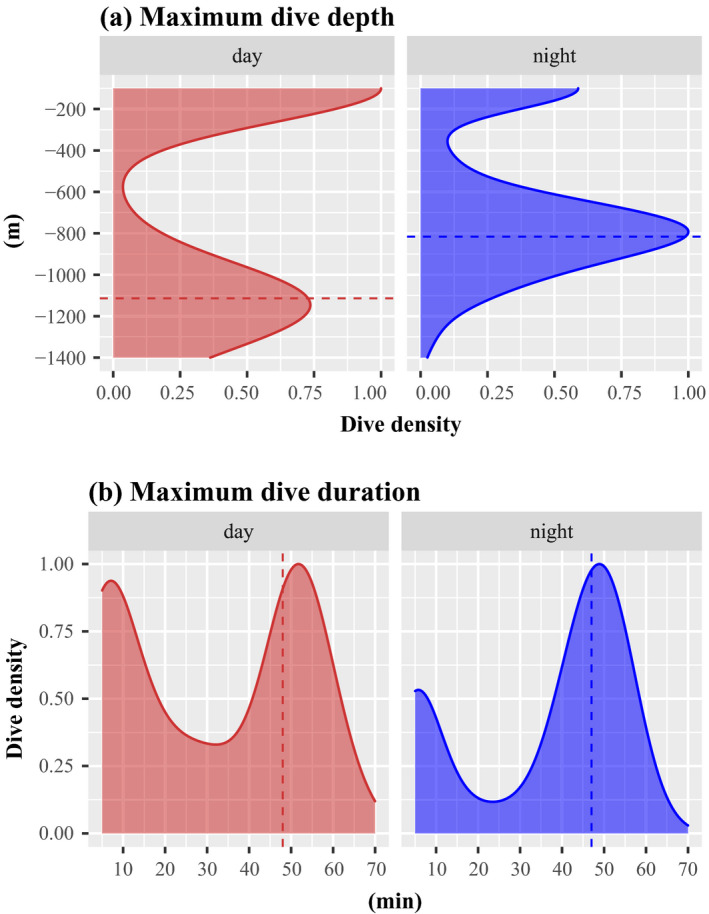
Kernel densities of the (a) maximum dive depth and the (b) maximum dive duration according to day (in red) and night (in blue)

A total of 500 maximum dive depths and 529 maximum dive durations were recorded from the eight whales equipped with SPLASH10 tags. The distributions of the dive depths were bimodal with mainly shallow (<500 m during the day and < 400 m at night) and deep dives, that is, between 600 and 1,400 m during the day and 400 and 1,400 m at night (Figure 7a). When looking at the deep dives (>200 m), a diel pattern was observed for the maximum dive depth, with significantly deeper dives during the day (mean: 1,146 m) compared to night‐time dives (mean: 816 m, Kruskal–Wallis test, *p* < 0.001)—See Figure 7a,b.

Similarly, the distributions of the dive durations were also bimodal, with short (<30 min during the day and <25 min at night) and long dives (between 30 and 70 min during the day and 25 and 70 min at night, Figure 7b). Dive durations lasted on average 34 min (Figure 7b). Twenty percent of the dives were short and lasted less than 10 min, and 45% were long (between 40 and 60 min). However, there was no significant difference in terms of dive duration between day (mean = 31 min) and night (mean = 35 min, Kruskal–Wallis test, *p* = 0.9322).

## 4 DISCUSSION

Using a combination of tracking data, state‐of‐the‐art ocean products, and an innovative machine learning approach, our study is the first to shed light on the resident behavior and seasonal patterns of sperm whales off Mauritius Island and to provide maps of their potential distribution that will support conservation planning.

### 4.1 High‐use area in Mauritius waters

Sperm whales disperse widely in all ocean basins, and their global abundance estimate is in the hundreds of thousands (Whitehead, 2002). Results from our limited sample size from a localized population in the south‐west Indian Ocean may not be representative of the behavior of all sperm whales but fill a critical gap in our understanding of this deep diving species. The satellite tracked individuals highlighted two critical hot spots close to Mauritius as well as a migratory route between Mauritius and Rodrigues. Among the 21 sperm whales satellite tracked, 14 remained in close proximity to Mauritius up to a maximum of 107 days. The Mascarene Islands (Reunion and Mauritius Islands) have previously been identified as a suitable habitat for this species (Mannocci, Laran, et al., 2014), using aerial survey data. Here, satellite tracking data have allowed both resident and migratory movements of individually tracked whales to be described in this area and quantified. Although the time spent west of Mauritius varied across individuals, the kernel densities showed two clear hot spots located west and south‐west of Mauritius. These two core areas might correspond to a breeding and a nursery ground during the wet and dry season, respectively. Despite mature males being observed from September to June in Mauritius waters, a larger proportion of mature males is seen between October and December in one of the highlighted core areas, while more calves are mostly observed between March and April in the second one (M. Vely, unpublished data). The 16 months gestation period of this species (Ohsumi, 1965) and a previous study showing that conception takes place in the austral summer south‐east of South Africa (Findlay & Best, 2016) together with observations of sperm whales giving birth in April (Gambell, 1966) reinforce the importance of these potential breeding and nursery habitats in Mauritius waters.

### 4.2 Seasonal migratory patterns

Although the tracked sperm whales showed a strong site fidelity to Mauritius waters, a significant proportion of the individuals (40%) left the island's coastal waters to perform a short migration towards Rodrigues during the wet season. These migrant whales were all mature females except one, and in 2018, 70% of the tracked whales showed a surprisingly synchronized departure from Mauritius in mid‐December. These whales belong to two separate clans which are frequently observed interacting with each other (Sarano & Sarano, 2017). Similarly to the Atlantic, the genetic structure in the Indian Ocean is mostly attributed to geographic philopatry (Alexander et al., 2016), which could partly explain the substantial difference observed between males and females in our study (Engelhaupt et al., 2009).

In addition to social connections, environmental drivers might also explain such a migratory behavior. As the whales seemed to move into cooler areas, it is possible that an increase in temperature in Mauritius waters may have impacted them either directly, by affecting their physiology (i.e., capacity to dissipate excess heat), or indirectly by impacting the distribution of their prey. Unfortunately, direct data on prey distribution were not available for this region, and proxies of micronekton biomass via mid‐trophic level models (e.g., SEAPODYM) did not show temporal differences that could explain the apparent abandonment of coastal areas near Mauritius. Given that female sperm whales generally congregate into large social groups (Best & Folkens, 2007), their synchronized departure could also be related to their social structure and the contrasting behavior between males and females. Sperm whales are considered to be income breeders (Oftedal, 1997), and a behavioral dichotomy is generally observed between both sexes. In an Australian sperm whale population, Irvine et al. (2017) have shown that the males are present all year round, whereas the females are mostly seen between April–May and September–November, suggesting a migratory behavior similar to the one found in our study. Although some tracked females did not seem to have left Mauritius, it could simply be due to the relatively short tracking duration for these whales. The limited sample size of this study reinforces the need to track more individuals over the entire annual cycle to clarify the distribution and seasonal patterns of this sperm whale population. Although the majority of the mature males tracked from Mauritius remained in close proximity to the island, suggesting a resident behavior, several studies indicate that mature males move to higher latitudes before and after the breeding season (Mellinger et al., 2004; Whitehead, 2018; Wong & Whitehead, 2014). Accordingly, the only male that left Mauritius headed southward in a straight line. This male may have headed towards Crozet or Kerguelen Archipelagos, which are famous hot spots for this species where large males are regularly observed feeding on Patagonian toothfish from longliners (Tixier et al., 2019).

Even though sperm whales are occasionally found in coastal waters, they must be considered pelagic animals that forage on ephemeral prey resources over large ocean scales (Kawakami, 1980). The variable location of their prey resources may translate into seasonal foraging movements to maintain fitness. But to date, little is known about what drives the movements of sperm whales. In particular, nothing is known about their feeding habits in the waters around Mauritius. Their restricted and sinuous movements close to the island, however, suggest that they are also feeding in these waters, likely on squid, their main prey resource (Kawakami, 1980). In this study, the sperm whales conducted shallower dives at night, but did not seem to change the duration of their dives. Davis et al. (2007) studied diurnal vertical migrations of sperm whale and squid in the California Gulf and found that the whales followed the vertical excursions of squids in shallower depths during the night (Stewart et al., 2013), which is in agreement with the diel pattern found in our study. Squids are often considered to be sensitive to temperatures at depth and the vertical movement patterns of the sperm whales observed in this study may be in reaction to changes in squid diel vertical migration (Gilly et al., 2006). Although 11 tags were deployed to record dive data, unfortunately data from a few dives were transmitted, preventing comparison of the diving behavior between seasons and between males and females. Deployment of acoustic tags with time depth recorders and 3D accelerometers could confirm whether the sperm whales are feeding in this area.

### 4.3 Predicted distribution and its conservation implications

An important finding from this study is that even a small sample of tracked whales can provide new and important insight into the physical and oceanographic factors that drive the movements of this deep diving species. This is mainly thanks to the novel method used here to compare models for detecting habitat selection using 14 different supervised machine learning algorithms and to generate site‐specific insight into sperm whales' behavior. Unlike the traditional regression methods commonly used in SDMs (e.g., GLM), the machine learning‐based approach used in this study has the ability to model complex polynomial relationships without relying on unrealistic assumptions (e.g., linearity) (Thessen, 2016). Using the ensemble approach, we also provide a new way to combine predictions from several algorithms, which is to date rarely used in spatial ecology. We therefore recommend to test a minimum of five different algorithms when trying to predict animal's distribution, in order to capture the more complex relationships between a species’ occurrence and its environment, and therefore increase the predictive power of the model to get the most reliable predictions despite limited sample sizes. Our results show a strong seasonal pattern with more dispersed movements during the wet season (December–March) and affinity to contrasting environmental predictors according to the season. Our best model during the wet season showed the strongest affinity for SSH, which is in agreement with a previous study that showed higher sperm whales densities in areas of higher sea level anomalies (Mannocci, Laran, et al., 2014). This suggests that sperm whales are likely looking for enriched pelagic waters that could be associated with mesoscale features (i.e., eddies, fronts) when departing from Mauritius. However, we did not find any direct relationship between the whales' tracks and eddies or fronts east of Mauritius during the wet season, probably due to the relatively low mesoscale activity in the Mascarene compared to the Mozambique Channel, where sperm whales encounter rates are much higher (Mannocci, Laran, et al., 2014). During the dry season, the most important predictor was the bottom temperature followed by the bathymetry. This highlighted affinity for particular areas close to Mauritius that are likely associated with higher prey densities in colder waters at certain depths.

In our study, the habitat for sperm whales extended over a restricted latitudinal band (19.5–22°S), which contrasts with previous studies showing north–south migrations (Findlay & Best, 2016; Whitehead et al., 2008). During the dry season, the predicted distribution was limited to coastal waters of Mauritius and Reunion Islands, reinforcing the need to implement conservation measures in these areas, that is, promote reserve designation and extend the actual MPAs. Currently, Mauritius has eight MPAs including two marine parks and six areas declared as fishing reserves. They are, however, relatively small (between 3.5 and 63.4 km^2^) and confined to areas close to shore (Francis et al., 2002). Data on animal distribution are often lacking when designing MPAs, and findings like ours are therefore essential to support conservation planning. Our results could also contribute to the regulation of the whale watching industry, which is omnipresent in such touristic areas. Restricting disturbance of animals is of particular importance at breeding sites like Mauritius coastal waters. Rather than static and sometimes inadequate MPAs, here we recommend designing dynamic MPAs based on the seasonal prediction maps of the whales (Maxwell et al., 2015). In addition to filling a gap in our knowledge about the movements and habitats of sperm whales in the south‐western Indian Ocean, our study will contribute to the implementation of concrete conservation measures in the waters of Mauritius and Reunion by clearly delineating the breeding and foraging grounds of this vulnerable species. Our findings are of particular importance in the Indian Ocean, where regional assessments are still lacking despite the presence of sperm whales (Huijser et al., 2020; Laran, Authier, et al., 2017). Our results should therefore supplement existing sperm whale's records available from international databases like the Global Biodiversity Information Facility (GBIF) to support regional assessments.

## CONFLICT OF INTEREST

The authors declare no conflict of interest.

## AUTHOR CONTRIBUTIONS

**Philippine Chambault:** Formal analysis (lead); methodology (lead); writing – original draft (lead); writing – review and editing (equal). **Sabrina Fossette:** Investigation (equal); methodology (equal); supervision (equal); writing – review and editing (equal). **Mads Peter Heide‐Jørgensen:** Conceptualization (equal); investigation (equal); methodology (equal); resources (equal); supervision (equal); validation (equal); writing – review and editing (equal). **Daniel Jouannet:** Investigation (equal); methodology (equal); project administration (equal); writing – review and editing (equal). **Michel Vély:** Conceptualization (equal); data curation (equal); funding acquisition (lead); investigation (equal); methodology (equal); project administration (lead); supervision (equal); validation (equal); writing – review and editing (equal).

## Supporting information

Figure S1

Figure S2

Supplementary Material

## Data Availability

Data are available from the Dryad Digital Repository: https://datadryad.org/stash/metadata_entry_pages/find_or_create?resource_id=95550.

## References

[ece37154-bib-0001] Alexander, A. , Steel, D. , Hoekzema, K. , Mesnick, S. L. , Engelhaupt, D. , Kerr, I. , Payne, R. , & Baker, C. S. (2016). What influences the worldwide genetic structure of sperm whales (*Physeter macrocephalus*)? Molecular Ecology, 25, 2754–2772. 27037911 10.1111/mec.13638

[ece37154-bib-0002] Andrews, R. D. , Baird, R. W. , Calambokidis, J. , Goertz, C. E. C. , Gulland, F. M. D. , Heide‐Jorgensen, M.‐P. , Hooker, S. K. , Johnson, M. , Mate, B. , Mitani, Y. , Nowacek, D. P. , Owen, K. , Quakenbush, L. T. , Raverty, S. , Robbins, J. , Schorr, G. S. , Shpak, O. V. , Townsend, F. I. , Uhart, M. , … Zerbini, A. N. (2019). Best practice guidelines for cetacean tagging. Journal of Cetacean Research and Management, 20(1), 27–66. 10.47536/jcrm.v20i1.237

[ece37154-bib-0003] Austin, M. P. (2002). Spatial prediction of species distribution: An interface between ecological theory and statistical modelling. Ecological Modelling, 157, 101–118. 10.1016/S0304-3800(02)00205-3

[ece37154-bib-0004] Berger, K. M. , & Gese, E. M. (2007). Does interference competition with wolves limit the distribution and abundance of coyotes? Journal of Animal Ecology, 76, 1075–1085. 10.1111/j.1365-2656.2007.01287.x 17922704

[ece37154-bib-0005] Best, P. B. , & Folkens, P. A. (2007). Whales and dolphins of the Southern African subregion. Cambridge University Press.

[ece37154-bib-0006] Biggs, D. , Leben, R. , & Ortega‐Ortiz, J. (2000). Ship and satellite studies of mesoscale circulation and sperm whale habitats in the Northeast Gulf of Mexico during GulfCet II. Gulf of Mexico Science, 18(1). 10.18785/goms.1801.02

[ece37154-bib-0007] Cañadas, A. , Sagarminaga, R. , & García‐Tiscar, S. (2002). Cetacean distribution related with depth and slope in the Mediterranean waters off southern Spain. Deep Sea Research Part I: Oceanographic Research Papers, 49, 2053–2073. 10.1016/S0967-0637(02)00123-1

[ece37154-bib-0008] Chambault, P. , Dalleau, M. , Nicet, J.‐B. , Mouquet, P. , Ballorain, K. , Jean, C. , Ciccione, S. , & Bourjea, J. (2020). Contrasted habitats and individual plasticity drive the fine scale movements of juvenile green turtles in coastal ecosystems. Movement Ecology, 8, 1 10.1186/s40462-019-0184-2 31921423 PMC6947949

[ece37154-bib-0009] Christal, J. , Whitehead, H. , & Lettevall, E. (2011). Sperm whale social units: Variation and change. Canadian Journal of Zoology, 76(8), 1431–1440.

[ece37154-bib-0010] Davis, R. W. , Jaquet, N. , Gendron, D. , Markaida, U. , Bazzino, G. , & Gilly, W. (2007). Diving behavior of sperm whales in relation to behavior of a major prey species, the jumbo squid, in the Gulf of California, Mexico. Marine Ecology Progress Series, 333, 291–302. 10.3354/meps333291

[ece37154-bib-0011] Elith, J. , H. Graham, C. , P. Anderson, R. , Dudík, M. , Ferrier, S. , Guisan, A. , J. Hijmans, R. , Huettmann, F. , R. Leathwick, J. , Lehmann, A. , Li, J. , G. Lohmann, L. , A. Loiselle, B. , Manion, G. , Moritz, C. , Nakamura, M. , Nakazawa, Y. , McC. M. Overton, J. , Townsend Peterson, A. , … E. Zimmermann, N. (2006). Novel methods improve prediction of species' distributions from occurrence data. Ecography, 29, 129–151. 10.1111/j.2006.0906-7590.04596.x

[ece37154-bib-0012] Elith, J. , & Leathwick, J. R. (2009). Species distribution models: Ecological explanation and prediction across space and time. Annual Review of Ecology, Evolution, and Systematics, 40, 677–697. 10.1146/annurev.ecolsys.110308.120159

[ece37154-bib-0013] Engelhaupt, D. , Hoelzel, A. R. , Nicholson, C. , Frantzis, A. , Mesnick, S. , Gero, S. , Whitehead, H. , Rendell, L. , Miller, P. , Stefanis, R. D. , Cañadas, A. , Airoldi, S. , & Mignucci‐Giannoni, A. A. (2009). Female philopatry in coastal basins and male dispersion across the North Atlantic in a highly mobile marine species, the sperm whale (*Physeter macrocephalus*). Molecular Ecology, 18, 4193–4205. 10.1111/j.1365-294X.2009.04355.x 19769692

[ece37154-bib-0014] Findlay, K. P. , & Best, P. B. (2016). Distribution and seasonal abundance of large cetaceans in the Durban whaling grounds off KwaZulu‐Natal, South Africa, 1972–1975. African Journal of Marine Science, 38, 249–262. 10.2989/1814232X.2016.1191042

[ece37154-bib-0015] Francis, J. , Nilsson, A. , & Waruinge, D. (2002). Marine Protected Areas in the Eastern African Region. How Successful Are They? Ambi, 31, 503–511. 10.1579/0044-7447-31.7.503 12572815

[ece37154-bib-0016] Gambell, R. (1966). Foetal growth and the breeding season of sperm whales. Norsk Hvalfangst‐tidende.

[ece37154-bib-0017] Gannier, A. , Drouot, V. , & Goold, J. C. (2002). Distribution and relative abundance of sperm whales in the Mediterranean Sea. Marine Ecology Progress Series, 243, 281–293. 10.3354/meps243281

[ece37154-bib-0018] Gannier, A. , & Praca, E. (2007). SST fronts and the summer sperm whale distribution in the north‐west Mediterranean Sea. Journal of the Marine Biological Association of the United Kingdom, 87, 187–193. 10.1017/S0025315407054689

[ece37154-bib-0019] Gilly, W. F. , Markaida, U. , Baxter, C. H. , Block, B. A. , Boustany, A. , Zeidberg, L. , Reisenbichler, K. , Robison, B. , Bazzino, G. , & Salinas, C. (2006). Vertical and horizontal migrations by the jumbo squid Dosidicus gigas revealed by electronic tagging. Marine Ecology Progress Series, 324, 1–17. 10.3354/meps324001

[ece37154-bib-0020] Hattab, T. , Garzón‐López, C. X. , Ewald, M. , Skowronek, S. , Aerts, R. , Horen, H. , Brasseur, B. , Gallet‐Moron, E. , Spicher, F. , Decocq, G. , Feilhauer, H. , Honnay, O. , Kempeneers, P. , Schmidtlein, S. , Somers, B. , Kerchove, R. V. D. , Rocchini, D. , & Lenoir, J. (2017). A unified framework to model the potential and realized distributions of invasive species within the invaded range. Diversity and Distributions, 23, 806–819. 10.1111/ddi.12566

[ece37154-bib-0021] Heide‐Jorgensen, M. P. , Kleivane, L. , Oien, N. , Laidre, K. L. , & Jensen, M. V. (2001). A new technique for deploying satellite transmitters on baleen whales: Tracking a blue whale (*Balaenoptera musculus* in the North Atlantic. Marine Mammal Science, 17, 949–954. 10.1111/j.1748-7692.2001.tb01309.x

[ece37154-bib-0022] Holt, S. J. (1983). The Indian Ocean Whale Sanctuary. Ambio, 12, 345–347.

[ece37154-bib-0023] Hooker, S. K. , & Gerber, L. R. (2004). Marine reserves as a tool for ecosystem‐based management: The potential importance of megafauna. BioScience, 54, 27–39. 10.1641/0006-3568(2004)054[0027:MRAATF]2.0.CO;2

[ece37154-bib-0024] Huijser, L. A. E. , Estrade, V. , Webster, I. , Mouysset, L. , Cadinouche, A. , & Dulau‐Drouot, V. (2020). Vocal repertoires and insights into social structure of sperm whales (*Physeter macrocephalus*) in Mauritius, southwestern Indian Ocean. Marine Mammal Science, 36, 638–657.

[ece37154-bib-0025] Irvine, L. G. , Thums, M. , Hanson, C. E. , McMahon, C. R. , & Hindell, M. A. (2017). Quantifying the energy stores of capital breeding humpback whales and income breeding sperm whales using historical whaling records. Royal Society Open Science, 4, 160290 10.1098/rsos.160290 28405350 PMC5383807

[ece37154-bib-0026] Iturbide, M. , Bedia, J. , Herrera, S. , del Hierro, O. , Pinto, M. , & Gutiérrez, J. M. (2015). A framework for species distribution modelling with improved pseudo‐absence generation. Ecological Modelling, 312, 166–174. 10.1016/j.ecolmodel.2015.05.018

[ece37154-bib-0027] Jacques, C. N. , Jenks, J. A. , & Klaver, R. W. (2009). Seasonal movements and home‐range use by female pronghorns in sagebrush‐steppe communities of Western South Dakota. Journal of Mammalogy, 90, 433–441. 10.1644/07-MAMM-A-395.1

[ece37154-bib-0028] Jaquet, N. (1996). How spatial and temporal scales influence understanding of Sperm Whale distribution: A review. Mammal Review, 26, 51–65. 10.1111/j.1365-2907.1996.tb00146.x

[ece37154-bib-0029] Jaquet, N. , Dawson, S. , & Slooten, E. (2000). Seasonal distribution and diving behaviour of male sperm whales off Kaikoura: Foraging implications. Canadian Journal of Zoology, 78, 407–419. 10.1139/z99-208

[ece37154-bib-0030] Kawakami, T. (1980). A review of sperm whale food (pp. 199–218). The Scientific Reports of the Whales Research Institute.

[ece37154-bib-0031] Kie, J. G. (2013). A rule‐based ad hoc method for selecting a bandwidth in kernel home‐range analyses. Animal Biotelemetry, 1, 13 10.1186/2050-3385-1-13

[ece37154-bib-0032] Lambert, C. , Mannocci, L. , Lehodey, P. , & Ridoux, V. (2014). Predicting cetacean habitats from their energetic needs and the distribution of their prey in two contrasted tropical regions. PLoS One, 9, e105958 10.1371/journal.pone.0105958 25162643 PMC4146581

[ece37154-bib-0033] Laran, S. , Authier, M. , Van Canneyt, O. , Dorémus, G. , Watremez, P. , & Ridoux, V. (2017). A comprehensive survey of pelagic megafauna: Their distribution, densities, and taxonomic richness in the tropical southwest Indian Ocean. Frontiers in Marine Science, 4, 139 10.3389/fmars.2017.00139

[ece37154-bib-0034] Laran, S. , & Drouot‐Dulau, V. (2007). Seasonal variation of striped dolphins, fin‐ and sperm whales' abundance in the Ligurian Sea (Mediterranean Sea). Journal of the Marine Biological Association of the United Kingdom, 87, 345–352. 10.1017/S0025315407054719

[ece37154-bib-0035] Laran, S. , Pettex, E. , Authier, M. , Blanck, A. , David, L. , Dorémus, G. , Falchetto, H. , Monestiez, P. , Van Canneyt, O. , & Ridoux, V. (2017). Seasonal distribution and abundance of cetaceans within French waters‐ Part I: The North‐Western Mediterranean, including the Pelagos sanctuary. Deep Sea Research Part II: Topical Studies in Oceanography, 141, 20–30. 10.1016/j.dsr2.2016.12.011

[ece37154-bib-0036] Lopez, R. , Malardé, J.‐P. , Royer, F. , & Gaspar, P. (2014). Improving Argos Doppler location using multiple‐model Kalman filtering. IEEE Transactions on Geoscience and Remote Sensing, 52, 4744–4755. 10.1109/TGRS.2013.2284293

[ece37154-bib-0037] Mannocci, L. , Catalogna, M. , Dorémus, G. , Laran, S. , Lehodey, P. , Massart, W. , Monestiez, P. , Van Canneyt, O. , Watremez, P. , & Ridoux, V. (2014). Predicting cetacean and seabird habitats across a productivity gradient in the South Pacific gyre. Progress in Oceanography, 120, 383–398. 10.1016/j.pocean.2013.11.005

[ece37154-bib-0038] Mannocci, L. , Laran, S. , Monestiez, P. , Dorémus, G. , Canneyt, O. V. , Watremez, P. , & Ridoux, V. (2014). Predicting top predator habitats in the Southwest Indian Ocean. Ecography, 37, 261–278. 10.1111/j.1600-0587.2013.00317.x

[ece37154-bib-0039] Maxwell, S. M. , Hazen, E. L. , Lewison, R. L. , Dunn, D. C. , Bailey, H. , Bograd, S. J. , Briscoe, D. K. , Fossette, S. , Hobday, A. J. , Bennett, M. , Benson, S. , Caldwell, M. R. , Costa, D. P. , Dewar, H. , Eguchi, T. , Hazen, L. , Kohin, S. , Sippel, T. , & Crowder, L. B. (2015). Dynamic ocean management: Defining and conceptualizing real‐time management of the ocean. Marine Policy, 58, 42–50. 10.1016/j.marpol.2015.03.014

[ece37154-bib-0040] Mellinger, D. K. , Stafford, K. M. , & Fox, C. G. (2004). Seasonal Occurrence of Sperm Whale (*Physeter Macrocephalus*) Sounds in the Gulf of Alaska, 1999–2001. Marine Mammal Science, 20, 48–62. 10.1111/j.1748-7692.2004.tb01140.x

[ece37154-bib-0041] Oftedal, O. T. (1997). Lactation in whales and dolphins: Evidence of divergence between baleen‐ and toothed‐species. Journal of Mammary Gland Biology and Neoplasia, 2, 205–230. 10882306 10.1023/a:1026328203526

[ece37154-bib-0042] Ohsumi, S. (1965). Reproduction of the sperm whale in the North‐West Pacific. Scientific Reports of the Whales Research Institute.

[ece37154-bib-0043] Perrin, W. F. , Würsig, B. , & Thewissen, J. G. M. (2009). Encyclopedia of marine mammals. Academic Press.

[ece37154-bib-0044] Pirotta, E. , Brotons, J. M. , Cerdà, M. , Bakkers, S. , & Rendell, L. E. (2020). Multi‐scale analysis reveals changing distribution patterns and the influence of social structure on the habitat use of an endangered marine predator, the sperm whale *Physeter macrocephalus* in the Western Mediterranean Sea. Deep Sea Research Part I: Oceanographic Research Papers, 155, 103169 10.1016/j.dsr.2019.103169

[ece37154-bib-0045] Pirotta, E. , Matthiopoulos, J. , MacKenzie, M. , Scott‐Hayward, L. , & Rendell, L. (2011). Modelling sperm whale habitat preference: A novel approach combining transect and follow data. Marine Ecology Progress Series, 436, 257–272. 10.3354/meps09236

[ece37154-bib-0046] Praca, E. , Gannier, A. , Das, K. , & Laran, S. (2009). Modelling the habitat suitability of cetaceans: Example of the sperm whale in the northwestern Mediterranean Sea. Deep Sea Research Part I: Oceanographic Research Papers, 56, 648–657. 10.1016/j.dsr.2008.11.001

[ece37154-bib-0047] R Core Team (2020). R: A language and environment for statistical computing. R Foundation for Statistical Computing Retrieved from http://www.R‐project.org/

[ece37154-bib-0048] Sarano, F. , & Sarano, M. (2017). Cartes d'identité des cachalots de l'île Maurice. Mauritius Marine Conservation Organization.

[ece37154-bib-0049] Schickele, A. , Leroy, B. , Beaugrand, G. , Goberville, E. , Hattab, T. , Francour, P. , & Raybaud, V. (2020). Modelling European small pelagic fish distribution: Methodological insights. Ecological Modelling, 416, 108902 10.1016/j.ecolmodel.2019.108902

[ece37154-bib-0050] Senay, S. D. , Worner, S. P. , & Ikeda, T. (2013). Novel three‐step pseudo‐absence selection technique for improved species distribution modelling. PLoS One, 8, e71218 10.1371/journal.pone.0071218 23967167 PMC3742778

[ece37154-bib-0051] Stewart, J. S. , Field, J. C. , Markaida, U. , & Gilly, W. F. (2013). Behavioral ecology of jumbo squid (*Dosidicus gigas*) in relation to oxygen minimum zones. Deep Sea Research Part II: Topical Studies in Oceanography, 95, 197–208. 10.1016/j.dsr2.2012.06.005

[ece37154-bib-0052] Taylor, B. , Baird, R. , Barlow, J. , Dawson, S. M. , Ford, J. , Mead, J. G. , Notarbartolo di Sciara, G. , Wade, P. , & Pitman, R. L. (2019). Physeter macrocephalus (amended version of 2008 assessment). The IUCN Red List of Threatened Species 2019: e.T41755A160983555. Available at https://www.iucnredlist.org/species/41755/160983555

[ece37154-bib-0053] Teloni, V. , Mark, J. P. , Patrick, M. J. O. , & Peter, M. T. (2008). Shallow food for deep divers: Dynamic foraging behavior of male sperm whales in a high latitude habitat. Journal of Experimental Marine Biology and Ecology, 354, 119–131. 10.1016/j.jembe.2007.10.010

[ece37154-bib-0054] Thessen, A. (2016). Adoption of machine learning techniques in ecology and earth science. One Ecosystem, 1, e8621 10.3897/oneeco.1.e8621

[ece37154-bib-0055] Tixier, P. , Welsford, D. C. , Lea, M.‐A. , Hindell, M. A. , Guinet, C. , Janc, A. , Richard, G. , Gasco, N. , Duhamel, G. , Arangio, R. , Villanueva, M. C. , Suberg, L. , & Arnould, J. P. Y. (2019). Fisheries interaction data suggest variations in the distribution of sperm whales on the Kerguelen Plateau In D. Welsford , J. Dell , & G. Duhamel (Eds.), The Kerguelen Plateau: Marine ecosystem and fisheries (pp. 259‐270). Proceedings of the Second Symposium. Australian Antarctic Division.

[ece37154-bib-0056] Vierod, A. D. T. , Guinotte, J. M. , & Davies, A. J. (2014). Predicting the distribution of vulnerable marine ecosystems in the deep sea using presence‐background models. Deep Sea Research Part II: Topical Studies in Oceanography, 99, 6–18. 10.1016/j.dsr2.2013.06.010

[ece37154-bib-0057] Virgili, A. , Authier, M. , Boisseau, O. , Cañadas, A. , Claridge, D. , Cole, T. , Corkeron, P. , Dorémus, G. , David, L. , Di‐Méglio, N. , Dunn, C. , Dunn, T. E. , García‐Barón, I. , Laran, S. , Lauriano, G. , Lewis, M. , Louzao, M. , Mannocci, L. , Martínez‐Cedeira, J. , … Ridoux, V. (2019). Combining multiple visual surveys to model the habitat of deep‐diving cetaceans at the basin scale. Global Ecology and Biogeography, 28, 300–314. 10.1111/geb.12850

[ece37154-bib-0058] Virgili, A. , Racine, M. , Authier, M. , Monestiez, P. , & Ridoux, V. (2017). Comparison of habitat models for scarcely detected species. Ecological Modelling, 346, 88–98. 10.1016/j.ecolmodel.2016.12.013

[ece37154-bib-0059] Wahlberg, M. (2002). The acoustic behaviour of diving sperm whales observed with a hydrophone array. Journal of Experimental Marine Biology and Ecology, 281, 53–62. 10.1016/S0022-0981(02)00411-2

[ece37154-bib-0060] Waring, G. T. , Hamazaki, T. , Sheehan, D. , Wood, G. , & Baker, S. (2001). Characterization of Beaked Whale (ziphiidae) and Sperm Whale (*physeter Macrocephalus*) Summer Habitat in Shelf‐Edge and Deeper Waters Off the Northeast U.S. Marine Mammal Science, 17, 703–717. 10.1111/j.1748-7692.2001.tb01294.x

[ece37154-bib-0061] Watkins, W. A. , Daher, M. A. , Dimarzio, N. A. , Samuels, A. , Wartzok, D. , Fristrup, K. M. , Gannon, D. P. , Howey, P. W. , Maiefski, R. R. , & Spradlin, T. R. (1999). Sperm whale surface activity from tracking by radio and satellite Tags1. Marine Mammal Science, 15, 1158–1180. 10.1111/j.1748-7692.1999.tb00883.x

[ece37154-bib-0062] Watwood, S. L. , Miller, P. J. O. , Johnson, M. , Madsen, P. T. , & Tyack, P. L. (2006). Deep‐diving foraging behaviour of sperm whales (*Physeter macrocephalus*). Journal of Animal Ecology, 75, 814–825. 10.1111/j.1365-2656.2006.01101.x 16689963

[ece37154-bib-0063] Whitehead, H. (2002). Estimates of the current global population size and historical trajectory for sperm whales. Marine Ecology Progress Series, 242, 295–304. 10.3354/meps242295

[ece37154-bib-0064] Whitehead, H. (2003). Sperm whales: Social evolution in the ocean. University of Chicago Press.

[ece37154-bib-0065] Whitehead, H. (2018). Sperm Whale: *Physeter macrocephalus* In B. Würsig , J. G. M. Thewissen , & K. M. Kovacs (Eds.), Encyclopedia of marine mammals (3rd ed., pp. 919–925). Academic Press.

[ece37154-bib-0066] Whitehead, H. , Coakes, A. , Jaquet, N. , & Lusseau, S. (2008). Movements of sperm whales in the tropical Pacific. Marine Ecology Progress Series, 361, 291–300. 10.3354/meps07412

[ece37154-bib-0067] Whitehead, H. , & Rendell, L. (2004). Movements, habitat use and feeding success of cultural clans of South Pacific sperm whales. Journal of Animal Ecology, 73, 190–196. 10.1111/j.1365-2656.2004.00798.x

[ece37154-bib-0068] Wong, S. N. P. , & Whitehead, H. (2014). Seasonal occurrence of sperm whales (*Physeter macrocephalus*) around Kelvin Seamount in the Sargasso Sea in relation to oceanographic processes. Deep Sea Research Part I: Oceanographic Research Papers, 91, 10–16. 10.1016/j.dsr.2014.05.001

[ece37154-bib-0069] Worton, B. J. (1989). Kernel methods for estimating the utilization distribution in home‐range studies. Ecology, 70, 164–168. 10.2307/1938423

