# Supplementary figures and images for: Predicting seasonal movements and distribution of the sperm whale using machine learning algorithms
Source: Ecol Evol. 2021 Jan 12;11(3):1432–45. doi: 10.1002/ece3.7154 (PMC7863674; doi:10.1002/ece3.7154)

**a) Wet season**

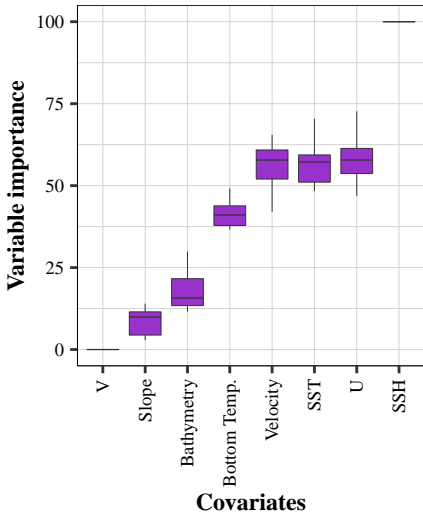

**b) Dry season**

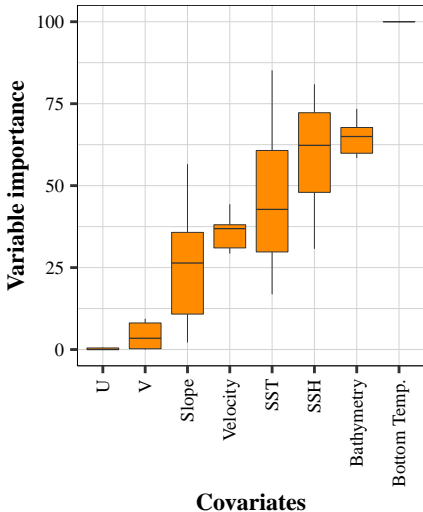

Supplement: Supplementary file 1 — Figure S1 [file ECE3-11-1432-s001.pdf]

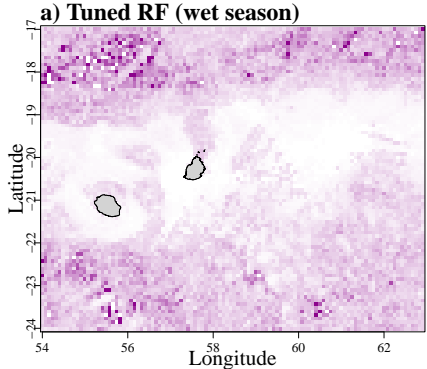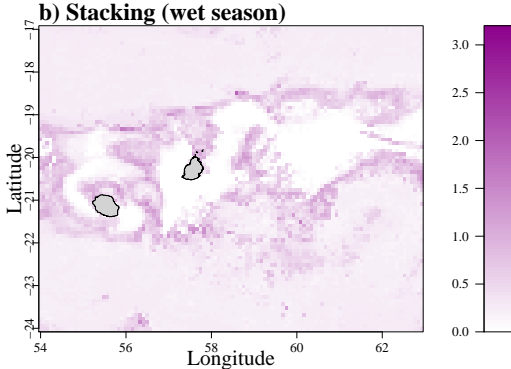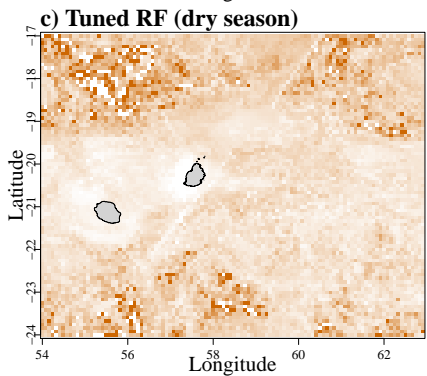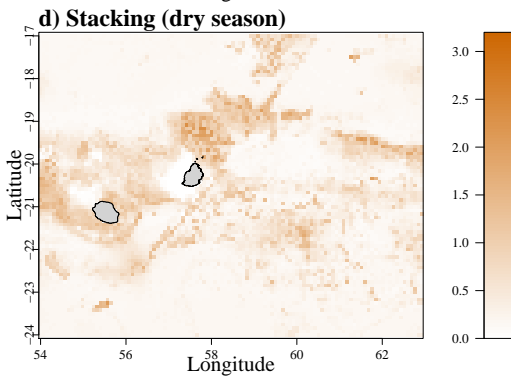

Supplement: Supplementary file 2 — Figure S2 [file ECE3-11-1432-s002.pdf]
