# Supplementary material for: Predicting seasonal movements and distribution of the sperm whale using machine learning algorithms
Source: Ecol Evol. 2021 Jan 12;11(3):1432–45. doi: 10.1002/ece3.7154 (PMC7863674; doi:10.1002/ece3.7154)
Supplement: Supplementary file 3 — Supplementary Material [file ECE3-11-1432-s003.docx]

**SUPPLEMENTARY INFORMATION**

**Figure S1.** Box plots of the covariates importance for the tuned random forest model and each season.

**Figure S2.** Maps of the coefficients of variation (expressed in percentage) during the (a, b) wet and (c, d) dry seasons calculated from the tuned random forest model (a, c) and the stacking method (b, d).
